# A Case Report on a Left Unicornuate Uterus With Communicating Right Rudimentary Horn Associated With Hematometra and Hematosalpinx

**DOI:** 10.7759/cureus.37959

**Published:** 2023-04-21

**Authors:** Shweta V Suryawanshi, Kanchan S Dwidmuthe

**Affiliations:** 1 Department of Obstetrics and Gynaecology, Narendra Kumar Prasadrao (NKP) Salve Institute of Medical Sciences and Research Centre, Nagpur, IND

**Keywords:** müllerian duct, hematosalpinx, rudimentary horn, congenital uterine anomalies, hematometra, unicornuate uterus

## Abstract

Congenital uterine anomalies (CUAs) or Müllerian duct anomalies are rare and can be either complete failure or partial failure in the development of the Mullerian duct, and they have a probability to result in a condition known as the unicornuate uterus. Partial development of one of the horns results in a rudimentary horn, which may be communicating consisting of category II A or noncommunicating consisting of category II B. This report illustrates a rare case of a 23-year-old female, unmarried, nulligravida, who presented to the outpatient department with chief complaints of acute abdominal pain and dysmenorrhea associated with an average menstrual flow. Pelvic ultrasound and magnetic resonance imaging (MRI) confirmed the diagnosis of a left unicornuate uterus with communicating right rudimentary horn associated with hematometra and hematosalpinx. As a treatment option, the surgical intervention mainly involved laparoscopic excision of the rudimentary horn and right salpingectomy that was performed by aspiration of blood from the rudimentary horn of around 25cc. Then, the right hydrosalpinx was removed, followed by right salpingectomy and excision of the rudimentary horn to reduce the risk of ectopic pregnancy having an incidence of 10% for which laparoscopic or robotic-assisted removal is preferable and practicable for young girls, compared with the open procedure. The patient adhered well to the surgical intervention.

## Introduction

Congenital uterine anomalies (CUAs) are not uncommon, having prevalence varying between 0.06% and 38%, which can be due to examination performed into different populations and consideration of different techniques for diagnosis [1]. However, in women with recurrent miscarriages, their prevalence ranges up to 16.7% [2]. An increased frequency of infertility, numerous first trimester abortions, labor that occurs prematurely, and fetal malposition are among the reproductive issues that affect about 25% of women with Müllerian duct defects [1].

Clinically, unicornuate uterus range from an incidental and symptomless finding to the complex pathology of the reproductive system often leading to infertility and abortions [3]. Menstrual cramps, also known as dysmenorrhea, are one of the most classic symptoms of CUAs and are also a prevalent complaint among teenage girls [4]. Either complete or partial failure in the development of the Müllerian duct leads to a unicornuate uterus class II by the American Society for Reproductive Medicine (ASRM) classification of Müllerian anomalies. A rudimentary horn is developed as a result of the incomplete development of one of the horns, and it may be communicating (II A) or noncommunicating (II B). The unicornuate uterus accounts for 10% of all fusion anomalies of the Müllerian duct [1,5]. This case report considered the American Society for Reproductive Medicine (ASRM) classification of Müllerian anomalies, which can be communicating (II A) or noncommunicating (II B).

However, appropriate diagnosis prior to surgical intervention is crucial for preventing future complications. The laparoscopic technique has been described in several cases, and this method seems to be effective because it is safe and efficient for the removal of rudimentary horns [6]. Laparoscopic approaches are advantageous because these procedures are minimally invasive, thus eliminating the need for prolonged hospital stays, providing faster recovery, along with better cosmetic outcomes when compared with similar results of laparotomy [7].

## Case presentation

**Figure 1:**
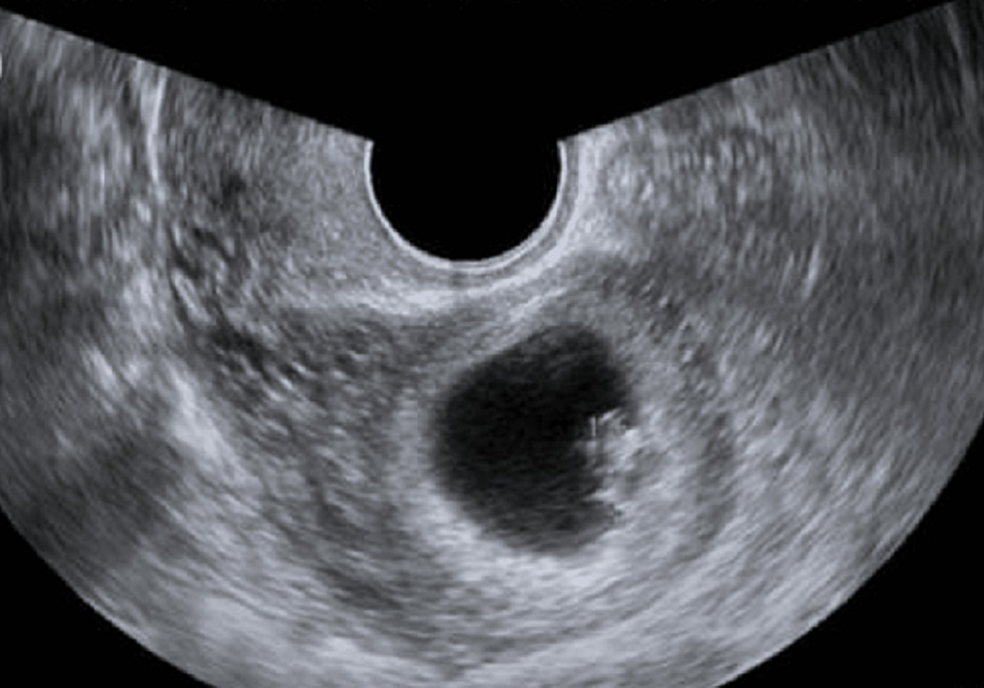
Sagittal section of the unicornuate uterus with left ovary.

A 23-year-old female, unmarried, nulligravida, presented to the outpatient department with chief complaints of acute abdominal pain and dysmenorrhea associated with normal menstrual flow. On examination, the patient was found vitally stable. On abdominal examination, there was rebound tenderness in the right lower quadrant with no rigidity. Furthermore, for diagnostic assessment, an ultrasound of the pelvis and abdomen was done, which showed the uterus tapering to one side, along with magnetic resonance imaging (MRI) of the pelvis, which demonstrated a curved and elongated uterus resembling banana-shaped external uterine contour, reduced uterine volume, asymmetric uterine configuration, and normal myometrial zonal anatomy that revealed evidence of unicornuate uterus with a communicating cavity of a rudimentary horn resulting from incomplete fusion of the ducts with functioning endometrium on the right side measuring approximately 6.5 × 4.1 × 4.6 cm (Figure 1).

However, attached to the left-sided unicornuate uterus on the fat-suppressed image (FAT-SAT), hyperintensity is noted in the abovementioned cavity of rudimentary horn in the endometrial cavity measuring 2.8 cm in thickness, which is suggestive of hemorrhagic collection. Also, there was evidence of a well-defined lesion in the cyst of the tubule on the right side of the pelvis that was about 2.8 cm wide that most likely originated from the right side of the fallopian tube. Moreover, both ovaries and the left fallopian tube appeared to be healthy. The diagnosis of a left unicornuate uterus with an associated right rudimentary horn, hematosalpinx, and hematometra was confirmed based on the aforementioned clinical features.

**Figure 2:**
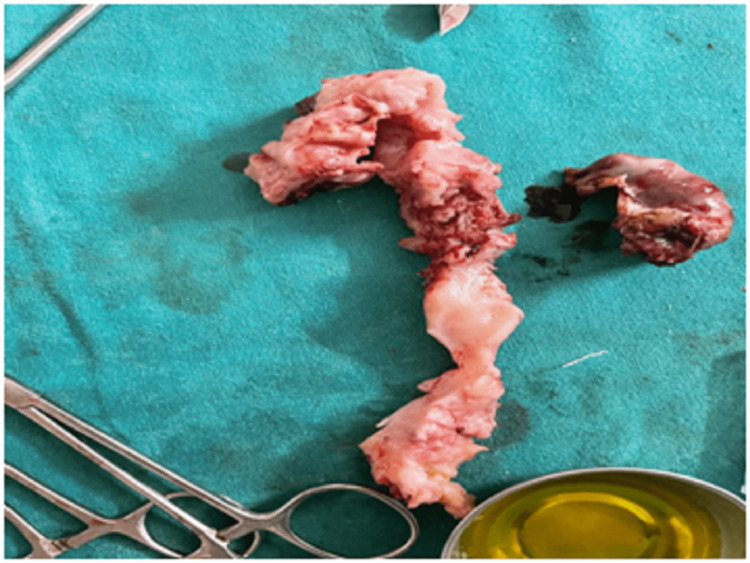
Specimen of the right rudimentary horn.

**Figure 3:**
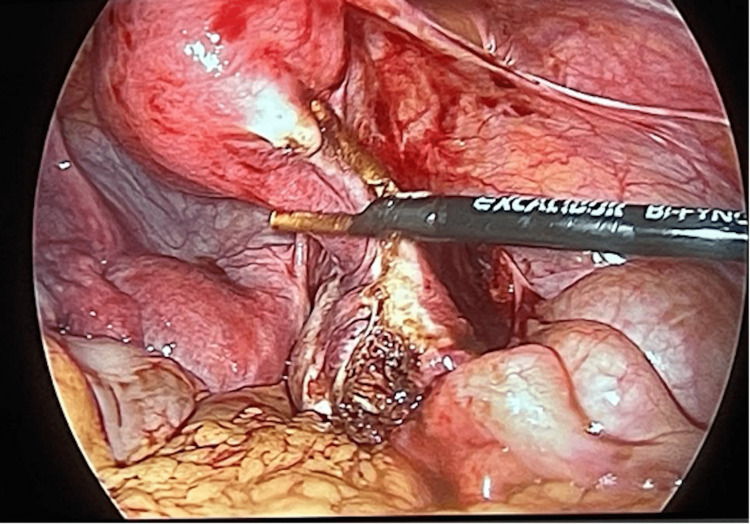
Uterus connected with the right cornuas.

The patient was advised and consented to surgical intervention that consisted of laparoscopic excision of the rudimentary horn and right salpingectomy, in which the patient was in a lithotomy position under general anesthesia, after which four-puncture laparoscopy involving a 10-mm infraumbilical port, a 10-mm suprapubic port, and two 5-mm suprapubic ports laterally in the right abdominal side and in the midline was performed. Around 25cc of blood from the rudimentary horn was aspirated; then, the right hydrosalpinx was removed, followed by right salpingectomy and excision of the rudimentary horn (Figure 2); and the uterus was connected with the right cornuas (Figure 3). The specimen retrieved was sent for pathological examination. The intraoperative findings revealed the right rudimentary horn with the right hydrosalpinx (Figure 2) and the appendix adherent to the right fallopian tube. The patient responded well to the surgical intervention, and as her recovery was exceptionally well without any complications, she was discharged the following day. The patient visited the outpatient department for a follow-up after four weeks following surgery and was symptom-free.

## Discussion

The incomplete development and fusion with the other side of Müllerian ducts result in the formation of a rudimentary horn with the unicornuate uterus. The American Society for Reproductive Medicine (ASRM) has given a classification of Müllerian duct abnormalities in which class II comprises the unicornuate uterus. This has been further divided into four sets of subdivisions wherein communicating is considered in II A, noncommunicating in II B, no cavity in II C, and no horn in II D [5,6]. In this presented case, the rudimentary horn’s cavity was functional and communicating with the uterus; hence, it would be categorized as class II A.

There is high variability in the clinical presentations of these rudimentary horns. Recently, in a study conducted by Jayasinghe et al. [8], 376 cases were reviewed, which were based on rudimentary horns, wherein they found that the majority of patients (78%) did not show any symptoms and were first appearing in the third decade of life. After menarche, women frequently reported abdominal pain or progressive dysmenorrhea, which was presumed to be due to endometriosis, hematosalpinx, and hematometra [6,8]. In this case, a left unicornuate uterus with a propagating rudimentary horn on the right side was associated with hematometra as well as hematosalpinx.

Pregnancy issues include malpresentation, frequent abortion, and premature labor, and women might also present with infertility. Early diagnosis is therefore crucial, but it is challenging because patients may remain asymptomatic [4,6,8]. However, in this case, the patient was symptomatic and reported early, leading to effective management.

In the present case, an MRI of the pelvis demonstrated a curved and elongated uterus resembling a banana-shaped external uterine contour, reduced uterine volume, asymmetric uterine configuration, and normal myometrial zonal anatomy that revealed evidence of unicornuate uterus with a communicating cavity of a rudimentary horn resulting from incomplete fusion of the ducts with functioning endometrium on the right side measuring approximately 6.5 × 4.1 × 4.6 cm. MRI, hysterosalpingogram, and sonohysterography, as well as two- and three-dimensional ultrasonography, are some of the techniques that aid in the assessment and evaluation of rudimentary horns. However, two-dimensional ultrasonography had a diagnostic sensitivity of only 26% [8]. It is, nonetheless, one of the usual modes of examination [9,10]. However, MRI is employed conventionally as it comes with an accuracy of 96% [11].

Previously, Atileh et al. [12] demonstrated a case of an ectopic ovary consisting of a unicornuate uterus with a rudimentary horn. Similarly, a case reported by Medeiros et al. [13] demonstrated that the laparoscopic technique for a unicornuate uterus with a noncommunicating horn is safe and effective. Additionally, a report of two cases given by Khanna et al. [14] described that the first-line weapon is a sonographic examination for the diagnosis of the unicornuate uterus along with rudimentary horn, followed by MRI, as it provides high-resolution images of the uterine body, fundus, and cervix and can assess the urinary tract for concomitant anomalies.

Given the complexity of the surgical treatment and the associated difficulties, diagnostic precision in imaging tests is critical in determining horn dimensions and the association of the unicornuate uterus with the horn. To enhance the results of surgical procedures, the diagnostic criteria for Müllerian abnormality in radiological investigations must be established. Therefore, in our case, laparoscopic removal is reported, which corresponds to previous studies as it has the advantages of posing minimal risk during the surgery and does not include a prolonged stay at the hospital and short recuperation period, as well as enhanced cosmetic outcomes [11].

## Conclusions

In conclusion, this highlights a rare case report of a 23-year-old female with a unicornuate uterus on the left side communicating with the rudimentary horn on the right side with hematometra and hematosalpinx. Additionally, it emphasizes the significance of early detection of the condition and progress for timely treatment to avoid any future consequences related to the condition. Also, it is critical to maintain a high degree of concern for vulnerable patients, especially women with renal, spinal, cloacal, and vertebra/anus/cardiac/trachea/esophagus/radius/renal/limb (VACTERL) abnormalities, and it is also important to carefully analyze the radiological images obtained from various radiological investigations. As a treatment option, a rudimentary horn should be excised to reduce the risk of ectopic pregnancy having an incidence of 10%, for which laparoscopic or robotic-assisted removal is feasible and preferred for young girls as it is less morbid and cosmetically more acceptable, compared with an open procedure.
